# Dynamics of Two-Link Musculoskeletal Chains during Fast Movements: Endpoint Force, Axial, and Shear Joint Reaction Forces

**DOI:** 10.3390/bioengineering10020240

**Published:** 2023-02-11

**Authors:** Andrea Biscarini

**Affiliations:** Department of Medicine and Surgery, University of Perugia, 06132 Perugia, Italy; andrea.biscarini@unipg.it; Tel.: +39-075-5858135

**Keywords:** biomechanics, kinetic chain, joint loads, fast movement, ballistic exercise

## Abstract

This study provides a dynamic model for a two-link musculoskeletal chain controlled by single-joint and two-joint muscles. The chain endpoint force, and the axial and shear components of the joint reaction forces, were expressed analytically as a function of the muscle forces or torques, the chain configuration, and the link angular velocities and accelerations. The model was applied to upper-limb ballistic push movements involving transverse plane shoulder flexion and elbow extension. The numerical simulation highlights that the shoulder flexion and elbow extension angular acceleration at the initial phase of the movement, and the elbow extension angular velocity and acceleration at the later phase of the movement, induce a proportional medial deviation in the endpoint force direction. The forearm angular velocity and acceleration selectively affect the value of the axial and shear components of the shoulder reaction force, depending on the chain configuration. The same goes for the upper arm and elbow. The combined contribution of the elbow extension angular velocity and acceleration may give rise to anterior shear force acting on the humerus and axial forearm traction force as high as 300 N. This information can help optimize the performance and estimate/control of the joint loads in ballistic sport activities and power-oriented resistance exercises.

## 1. Introduction

The planar system, composed of a fixed frame and two rigid bodies connected serially by ideal revolute joints controlled by torque actuators, is a reference model in robotics [1,2] and musculoskeletal biomechanics [3,4,5,6]. For the sake of brevity, throughout this paper, the term “chain” (or “serial chain”) is used to refer to this system, and the term “link” is used to refer to each rigid body component in the chain [1,2,3,4]. The mechanics of this ideal model have been characterized by the direct and inverse kinematic equations (establishing the relationship between the kinematic variables of the joints and those of the free endpoint of the chain), the corresponding direct and inverse static equations (determining the equilibrating joint torques necessary to maintain the chain equilibrium when an external force acts on the endpoint of the chain, and, inversely, the force exerted by the endpoint on a fixed external environment given the joint torques), the apparent endpoint and joint stiffness, and the closed-form dynamic equations (the dynamic equations that contain all the variables in explicit input–output form) [1,2,3,4].

The static equations for the two-link chain have been applied to the study of human limb biomechanics, considering the chain composed of the shoulder and elbow joints, the upper arm and the forearm-hand links, and the trunk as a fixed base or the corresponding lower limb chain [4]. Notably, previous static analyses established that the torque acting on the proximal joint (shoulder and hip) produces endpoint force along the pointing axis, which is the longitudinal axis of the distal link (forearm and lower leg), whereas the torque acting on the distal joint (elbow and knee) produces endpoint force along the radial axis, which is the axis connecting the proximal joint (shoulder and hip) with the chain endpoint (hand and foot) [4,7]. This information can potentially help therapists and athletic trainers analyze and optimize the technique of strength-training exercises, and estimate, even possibly control, the reaction forces acting on specific joint structures during these exercises [4].

Critically, the direct and inverse static equations can only be applied to isometric or quasi-static exercises, thus, limiting their potential application in exercise and sport sciences. During explosive motor actions (e.g., baseball pitch, team handball throw, tennis serve, volleyball spike, or shot put), the elbow extension reaches peak angular velocities between 1300 and 2300 °/s, and peak angular accelerations as high as 10^4^ ÷ 4·10^4^ °/s^2^ [8,9,10,11,12]. High elbow angular velocity (~850 °/s) can also be recorded during power-oriented exercises, such as the explosive bench press throws executed with low to moderate resistance (30–50% of one repetition maximum) [13]. In these fast dynamic conditions, the quasi-static computational approach is not applicable, and the dynamic equations of the chain should be used to determine the joint reaction forces and the relationship between the joint torques and the endpoint force.

The joints of a real musculoskeletal chain are driven by muscles that can span multiple joints, rather than ideal torque actuators. An ideal torque actuator solely produces torque about the joint axis with no effect on the joint reaction force [4]. In contrast, a muscle can produce torques among all the joints it crosses and can induce axial and shear joint reaction forces on each of these joints [14]. The selective determinations of the axial and shear reaction forces acting on a skeletal joint are fundamental to estimating how these forces are distributed among the different joint structures and periarticular tissues. The determination and control of such forces are some of the most crucial issues in musculoskeletal biomechanics [15,16,17,18,19,20,21,22,23].

To overcome the above limitations, this study provides a dynamic model for analysis of the fast movements of a planar two-link musculoskeletal chain driven by single-joint and two-joint muscles, modeled as force actuators attached to the links and the base. The study is aimed at deriving the analytical expression of the force exerted by the endpoint on the external environment as a function of the joint torques developed by muscles, the chain configuration, and the angular velocities and accelerations of the links. Numerical simulations were carried out considering a chain structure modelling the upper limb and dynamic chain configurations of particular interest in sports and exercise science. Further, the study investigated how the link angular velocities and accelerations contribute, in addition to the endpoint and muscle forces, to the development of the axial and shear components of the reaction forces acting on the joints. We hypothesize that the elbow and shoulder angular velocities and accelerations typically reached during ballistic sport movements can yield a relevant contribution to both the axial and shear reaction forces acting on the shoulder and elbow.

## 2. Materials and Methods

**Figure 1:**
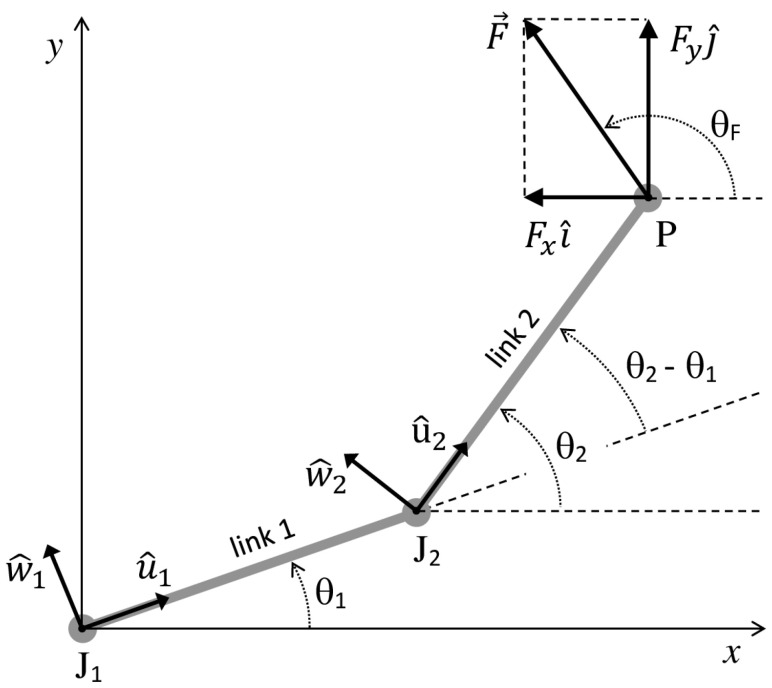
Mechanical diagram of the two-link chain with joint J_1_ and J_2_. The diagram includes the force $\vec{F}=F_{x}\hat{l}+F_{y}\hat{J}$ that the chain endpoint P exerts on the environment and the relevant angular quantities and unit vectors associated with each of the two links. The unit vectors of the x and y axis are $\hat{l}$ and $\hat{J}$, respectively.

We consider a planar musculoskeletal chain composed of 2 slender rigid links and ideal revolute joints (Figure 1). The proximal link of the chain (link 1) is articulated with the distal link (link 2) at joint J_2_ and with a fixed base (link 0) at joint J_1_. The free extremity of the distal link is referred to as the endpoint P of the chain. Single-joint muscles (connecting link 1 to link 2, or link 1 to the base) and biarticular muscles (connecting link 2 to the base) are modeled as linear force actuators. The following parameters are associated with each of the two links *i* (i=1, 2) of the chain: the mass $m_{i}$, the length $l_{i}$, the distance $l_{C_{i}}=\left|J_{i}C_{i}\right|$ from $J_{i}$ of the center of mass C*_i_* of the link, the link unit vector ${\hat{u}}_{i}$ pointing from $J_{i}$ along the link direction, the angle $\theta_{i}$ between ${\hat{u}}_{i}$ and the horizontal *x*-axis (the counterclockwise direction of rotation is considered positive), the moments of inertia $I_{J_{i}}$ about $J_{i}$, the angular velocity ${\;\vec{\omega }}_{i}={\dot{\theta }}_{i}\hat{k}$ and acceleration ${\dot{\vec{\omega }}}_{i}={\ddot{\theta }}_{i}\hat{k}$ ($\hat{k}$ is the unit vector normal to the plane of the chain), and the unit vector ${\hat{w}}_{i}=\hat{k}\times {\hat{u}}_{i}$.

The external forces acting on link 1 are the weight $m_{1}\vec{g}$ of the link applied at C_1_, the muscle forces $\underset{m}{\sum }{\vec{F}}_{12}^{m}$ and $\underset{m}{\sum }{\vec{F}}_{10}^{m}$ exerted on link 1 by the muscles connecting link 1 to link 2 and to link 0, respectively, and the joint reaction forces ${\vec{\phi }}_{1,0}$ and ${\vec{\phi }}_{1,2}$ exerted on link 1 by the base (link 0) and link 2, respectively. The external forces acting on link 2 are the weight $m_{2}\vec{g}$ of the link applied at C_2_, the muscle forces $\underset{m}{\sum }{\vec{F}}_{21}^{m}$ and $\underset{m}{\sum }{\vec{F}}_{20}^{m}$ exerted on link 2 by the muscles connecting link 2 to link 1 and to link 0, respectively, the joint reaction force ${\vec{\phi }}_{2,1}$ exerted on link 2 by link 1, and a contact external resistance $\vec{R}$ applied at the endpoint P of the chain. $R_{x}$ and $R_{y}$ denote the components of $\vec{R}$ relative to the *x-* and *y*-axis of the adopted global reference system $J_{1}$*xy* displayed in Figure 1. The above $\underset{m}{\sum }{\vec{F}}_{ij}^{m}$ summations are extended over all muscles joining link *i* to link *j*, and $P_{ij}^{m}$ will denote the point of application of ${\vec{F}}_{ij}^{m}$. The following relations hold: ${\vec{\phi }}_{i,j}=-{\vec{\phi }}_{j,i}$ and ${\vec{F}}_{ij}^{m}=-{\vec{F}}_{ji}^{m}$.

The moment equations for a planar chain composed of two slender links can be derived as a particular case of the general moment equations from Biscarini (2021) [14] for non-slender *n*-link chains driven by single-joint and multi-joint muscle actuators:

(1) $$\tau_{2}=m_{2}g\;l_{C_{2}}\cos\theta_{2}+R_{x}l_{2}{\sin\theta }_{2}-R_{y}l_{2}{\cos\theta }_{2}+I_{J_{2}}{\ddot{\theta }}_{2}\;+m_{2}l_{C_{2}}l_{1}\left[{\ddot{\theta }}_{1}\cos\left(\theta_{2}-\theta_{1}\right)+{\dot{\theta }}_{1}^{2}\sin\left(\theta_{2}-\theta_{1}\right)\right]$$

(2) $$\tau_{1}=\tau_{2}+m_{1}g\;l_{C_{1}}\cos\theta_{1}+m_{2}gl_{1}\cos\theta_{1}+R_{x}l_{1}{\sin\theta }_{1}-R_{y}l_{1}{\cos\theta }_{1}\;+\left(I_{J_{1}}+m_{2}l_{1}^{2}\right){\ddot{\theta }}_{1}+l_{1}m_{2}l_{C_{2}}\left[{\ddot{\theta }}_{2}\cos\left(\theta_{2}-\theta_{1}\right)-{\dot{\theta }}_{2}^{2}\sin\left(\theta_{2}-\theta_{1}\right)\right]$$

here, $\tau_{1}$ ($\tau_{2}$) is the torque developed about J_1_ (J_2_) by the distal to proximal forces of all single-joint and two-joint muscles crossing J_1_ (J_2_):

(3) $$\tau_{1}=\left(\underset{m}{\sum }J_{1}P_{10}^{m}\times {\vec{F}}_{10}^{m}+\underset{m}{\sum }J_{1}P_{20}^{m}\times {\vec{F}}_{20}^{m}\right)\cdot \hat{k}$$

(4) $$\tau_{2}=\left(\underset{m}{\sum }J_{2}P_{21}^{m}\times {\vec{F}}_{21}^{m}+\underset{m}{\sum }J_{2}P_{20}^{m}\times {\vec{F}}_{20}^{m}\right)\cdot \hat{k}$$

Moments are considered positive if they produce angular acceleration in a counterclockwise direction ($\hat{k}$ is the unit vector of the *z*-axis, normal to the plane of the chain). The joint reaction forces ${\vec{\phi }}_{1,2}=-{\vec{\phi }}_{2,1}$ and ${\vec{\phi }}_{0,1}=-{\vec{\phi }}_{0,1}$ can also be derived by [14]:

(5) $${\vec{\phi }}_{1,2}=m_{2}\vec{g}+R_{x}\hat{i}+R_{y}\hat{j}+\underset{m}{\sum }{\vec{F}}_{21}^{m}+\underset{m}{\sum }{\vec{F}}_{20}^{m}-m_{2}\left[l_{1}\left({\ddot{\theta }}_{1}{\hat{w}}_{1}-{\dot{\theta }}_{1}^{2}{\hat{u}}_{1}\right)+l_{C_{2}}\left({\ddot{\theta }}_{2}{\hat{w}}_{2}-{\dot{\theta }}_{2}^{2}{\hat{u}}_{2}\right)\right]$$

(6) $${\vec{\phi }}_{0,1}=m_{1}\vec{g}+m_{2}\vec{g}+R_{x}\hat{i}+R_{y}\hat{j}+\underset{m}{\sum }{\vec{F}}_{10}^{m}+\underset{m}{\sum }{\vec{F}}_{20}^{m}-m_{2}\left[l_{1}\left({\ddot{\theta }}_{1}{\hat{w}}_{1}-{\dot{\theta }}_{1}^{2}{\hat{u}}_{1}\right)+l_{C_{2}}\left({\ddot{\theta }}_{2}{\hat{w}}_{2}-{\dot{\theta }}_{2}^{2}{\hat{u}}_{2}\right)\right]\;-m_{1}l_{C_{1}}\left({\ddot{\theta }}_{1}{\hat{w}}_{1}-{\dot{\theta }}_{1}^{2}{\hat{u}}_{1}\right)$$

In Equations (1), (2), (5), and (6), different from the general case [14], we considered an external resistance $\vec{R}$ that only acts at the endpoint P of the chain; $\vec{R}$ is expressed in terms of its Cartesian components $R_{x}$ and $R_{y}$.

Equations (1) and (2) can be rewritten as a linear system in the unknowns $R_{x}$ and $R_{y}$:

(7) $$l_{1}{\sin\theta }_{1}R_{x}-l_{1}{\cos\theta }_{1}R_{y}\;=\tau_{1}-\tau_{2}-m_{1}g\;l_{C_{1}}\cos\theta_{1}-m_{2}gl_{1}\cos\theta_{1}-\left(I_{J_{1}}+m_{2}l_{1}^{2}\right){\ddot{\theta }}_{1}\;-l_{1}m_{2}l_{C_{2}}\left[{\ddot{\theta }}_{2}\cos\left(\theta_{2}-\theta_{1}\right)-{\dot{\theta }}_{2}^{2}\sin\left(\theta_{2}-\theta_{1}\right)\right]\;l_{2}{\sin\theta }_{2}R_{x}-l_{2}{\cos\theta }_{2}R_{y}=\tau_{2}-m_{2}g\;l_{C_{2}}\cos\theta_{2}-I_{J_{2}}{\ddot{\theta }}_{2}-m_{2}l_{C_{2}}l_{1}\left[{\ddot{\theta }}_{1}\cos\left(\theta_{2}-\theta_{1}\right)+{\dot{\theta }}_{1}^{2}\sin\left(\theta_{2}-\theta_{1}\right)\right]$$

According to Newton’s third law, the force $\vec{F}$ exerted by the endpoint P on the external environment that provides the external resistance $\vec{R}$ is given by $\vec{F}=-\vec{R}$. Thus, the $\vec{F}$ components are given by:

(8) $$\{\begin{array}{c} F_{x}=-R_{x}\\ F_{y}=-R_{y} \end{array}$$

In the case of external resistance of the inertial type, $\vec{R}$, and consequently $\vec{F}$, include the inertial force components related to the resistance mass acceleration. The solution of the Equation system (7) determines $R_{x}$ and $R_{y}$, then Equation (8) gives the analytical expressions of the and $F_{y}$ components of the force exerted by the chain endpoint:

(9) $$F_{x}=\frac{l_{2}{\cos\theta }_{2}}{l_{1}l_{2}\sin\left(\theta_{2}-\theta_{1}\right)}\left\{\tau_{1}-\tau_{2}-m_{1}g\;l_{C_{1}}\cos\theta_{1}-m_{2}gl_{1}\cos\theta_{1}-\left(I_{J_{1}}+m_{2}l_{1}^{2}\right){\ddot{\theta }}_{1}\;-l_{1}m_{2}l_{C_{2}}\left[{\ddot{\theta }}_{2}\cos\left(\theta_{2}-\theta_{1}\right)-{\dot{\theta }}_{2}^{2}\sin\left(\theta_{2}-\theta_{1}\right)\right]\right\}\;-\frac{l_{1}{\cos\theta }_{1}}{l_{1}l_{2}\sin\left(\theta_{2}-\theta_{1}\right)}\left\{\tau_{2}-m_{2}g\;l_{C_{2}}\cos\theta_{2}-I_{J_{2}}{\ddot{\theta }}_{2}\;-m_{2}l_{C_{2}}l_{1}\left[{\ddot{\theta }}_{1}\cos\left(\theta_{2}-\theta_{1}\right)+{\dot{\theta }}_{1}^{2}\sin\left(\theta_{2}-\theta_{1}\right)\right]\right\}$$

(10) $$F_{y}=\frac{l_{2}{\sin\theta }_{2}}{l_{1}l_{2}\sin\left(\theta_{2}-\theta_{1}\right)}\left\{\tau_{1}-\tau_{2}-m_{1}g\;l_{C_{1}}\cos\theta_{1}-m_{2}gl_{1}\cos\theta_{1}\;-\left(I_{J_{1}}+m_{2}l_{1}^{2}\right){\ddot{\theta }}_{1}\;-l_{1}m_{2}l_{C_{2}}\left[{\ddot{\theta }}_{2}\cos\left(\theta_{2}-\theta_{1}\right)-{\dot{\theta }}_{2}^{2}\sin\left(\theta_{2}-\theta_{1}\right)\right]\right\}\;-\frac{l_{1}{\sin\theta }_{1}}{l_{1}l_{2}\sin\left(\theta_{2}-\theta_{1}\right)}\left\{\tau_{2}-m_{2}g\;l_{C_{2}}\cos\theta_{2}-I_{J_{2}}{\ddot{\theta }}_{2}\;-m_{2}l_{C_{2}}l_{1}\left[{\ddot{\theta }}_{1}\cos\left(\theta_{2}-\theta_{1}\right)+{\dot{\theta }}_{1}^{2}\sin\left(\theta_{2}-\theta_{1}\right)\right]\right\}\;$$

The direction of $\vec{F}$ is determined by the following equation:

(11) $$\theta_{F}=\arctan\left(\frac{F_{y}}{F_{x}}\right)$$

where $\theta_{F}$ is the angle between $\vec{F}$ and the *x*-axis (Figure 1).

The axial and shear components of the joint reaction force ${\vec{\phi }}_{1,2}$ acting on J_2_ are determined by projecting ${\vec{\phi }}_{1,2}$ on the direction of ${\hat{u}}_{2}$ and ${\hat{w}}_{2}$, respectively:

(12) $${\left(\phi_{1,2}\right)}_{\mathrm{axial}}={\vec{\phi }}_{1,2}\cdot {\hat{u}}_{2}\;=R_{x}{\cos\theta }_{2}+\left(R_{y}-m_{2}g\right){\sin\theta }_{2}+\underset{m}{\sum }{\vec{F}}_{21}^{m}\cdot {\hat{u}}_{2}+\underset{m}{\sum }{\vec{F}}_{20}^{m}\cdot {\hat{u}}_{2}\;+m_{2}l_{C_{2}}{\dot{\theta }}_{2}^{2}+m_{2}l_{1}{\dot{\theta }}_{1}^{2}\cos\left(\theta_{2}-\theta_{1}\right)-m_{2}l_{1}{\ddot{\theta }}_{1}\sin\left(\theta_{2}-\theta_{1}\right)$$

(13) $${\left(\phi_{1,2}\right)}_{\mathrm{shear}}={\vec{\phi }}_{1,2}\cdot {\hat{w}}_{2}\;=-R_{x}{\sin\theta }_{2}+\left(R_{y}-m_{2}g\right){\cos\theta }_{2}+\underset{m}{\sum }{\vec{F}}_{21}^{m}\cdot {\hat{w}}_{2}+\underset{m}{\sum }{\vec{F}}_{20}^{m}\cdot {\hat{w}}_{2}\;-m_{2}l_{C_{2}}{\ddot{\theta }}_{2}-m_{2}l_{1}{\dot{\theta }}_{1}^{2}\sin\left(\theta_{2}-\theta_{1}\right)-m_{2}l_{1}{\ddot{\theta }}_{1}\cos\left(\theta_{2}-\theta_{1}\right)$$

Likewise, the axial and shear components of the joint reaction force ${\vec{\phi }}_{0,1}$ acting on J_1_ are determined by projecting ${\vec{\phi }}_{0,1}$ on the direction of ${\hat{u}}_{1}$ and ${\hat{w}}_{1}$, respectively:

(14) $${\left(\phi_{0,1}\right)}_{\mathrm{axial}}={\vec{\phi }}_{0,1}\cdot {\hat{u}}_{1}\;=R_{x}{\cos\theta }_{1}+\left(R_{y}-m_{1}g-m_{2}g\right){\sin\theta }_{1}+\underset{m}{\sum }{\vec{F}}_{10}^{m}\cdot {\hat{u}}_{1}+\underset{m}{\sum }{\vec{F}}_{20}^{m}\cdot {\hat{u}}_{1}+\left(m_{1}l_{C_{1}}+m_{2}l_{1}\right){\dot{\theta }}_{1}^{2}\;+m_{2}l_{C_{2}}{\dot{\theta }}_{2}^{2}\cos\left(\theta_{2}-\theta_{1}\right)+m_{2}l_{C_{2}}{\ddot{\theta }}_{2}\sin\left(\theta_{2}-\theta_{1}\right)$$

(15) $${\left(\phi_{0,1}\right)}_{\mathrm{shear}}={\vec{\phi }}_{0,1}\cdot {\hat{w}}_{1}\;=-R_{x}{\sin\theta }_{1}+\left(R_{y}-m_{1}g-m_{2}g\right){\cos\theta }_{1}+\underset{m}{\sum }{\vec{F}}_{10}^{m}\cdot {\hat{w}}_{1}+\underset{m}{\sum }{\vec{F}}_{20}^{m}\cdot {\hat{w}}_{1}-\left(m_{1}l_{C_{1}}+m_{2}l_{1}\right){\ddot{\theta }}_{1}\;+m_{2}l_{C_{2}}{\dot{\theta }}_{2}^{2}\sin\left(\theta_{2}-\theta_{1}\right)-m_{2}l_{C_{2}}{\ddot{\theta }}_{2}\cos\left(\theta_{2}-\theta_{1}\right)$$

In the following numerical simulation, $F_{x}$, $F_{y}$, and $\theta_{F}$ (given by Equations (9)–(11)) are regarded as functions of the 8 angular and torque variables $\tau_{1},\;\tau_{2},{\;\theta }_{1},\;\theta_{2},\;{\dot{\theta }}_{1},{\dot{\theta }}_{2},{\ddot{\theta }}_{1},$ and ${\ddot{\theta }}_{2}$. To highlight the effect of these variables, the weight of the links is neglected (this corresponds to chain movements in the horizontal plane) and the geometric and inertial quantities $l_{1},\;l_{2},\;l_{C_{1}},\;l_{C_{2}},\;m_{1},\;m_{2},\;I_{J_{1}},$ and $I_{J_{2}}$ are considered fixed parameters. Their values were retrieved from the anatomic data reported by Winter [24], considering the chain composed of the shoulder (S) and elbow (E) joints (J_1_ = S, J_2_ = E), the upper arm (link 1) and the forearm hand (link 2), and the stationary trunk (fixed base). With this musculoskeletal chain, $\tau_{1}$ and $\tau_{2}$ are the shoulder and elbow muscle torque, respectively. An upper limb push movement in the transverse plane will be specifically analyzed. By convention, positive (negative) values of $\tau_{1}$ reflect a shoulder flexion (extension) muscle torque in that plane, while positive (negative) values of $\tau_{2}$ reflect an elbow flexion (extension) muscle torque.

The values of $F_{x}$, $F_{y}$, and $\theta_{F}$ were analyzed for specific subsets of values of $\tau_{1},\;\tau_{2},{\;\theta }_{1},\;\theta_{2},\;{\dot{\theta }}_{1},{\dot{\theta }}_{2},{\ddot{\theta }}_{1},$ and ${\ddot{\theta }}_{2}$ with physiological meaning and specific interest in human movement and physical exercise:

- Condition 1. Effect of the upper arm angular acceleration ${\ddot{\theta }}_{1}$ (which coincides with the shoulder flexion angular acceleration) and the shoulder flexion muscle torque $\tau_{1}$ ($\tau_{1}>0,\;\tau_{2}\sim 0$) at the beginning phase of an upper limb push movement, with the forearm maintained in forward direction ($\theta_{1}=-30{}^{\circ},\;\theta_{2}=90{}^{\circ},\;{\dot{\theta }}_{1}={\dot{\theta }}_{2}={\ddot{\theta }}_{2}=0$). In this condition, the elbow extension angular acceleration is equal in magnitude to the shoulder flexion angular acceleration.
- Condition 2. Effect of the forearm angular velocity ${\dot{\theta }}_{2}$ and acceleration ${\ddot{\theta }}_{2}$, and the elbow extension muscle toque $\tau_{2}$ ($\tau_{2}<0,\;\tau_{1}\sim 0$), at the subsequent phase of the movement, when $\theta_{1}=60{}^{\circ},\;\theta_{2}=120{}^{\circ}$, and the upper arm angular velocity and acceleration are negligible compared to those of the forearm. In this condition, ${\dot{\theta }}_{2}$ and ${\ddot{\theta }}_{2}$ coincide approximately with the elbow angular velocity and acceleration.

In these two conditions, the contributions of the link angular velocities and accelerations to the axial and shear components of the joint reaction forces ${\vec{\phi }}_{0,1}$ and ${\vec{\phi }}_{1,2}$ were determined by Equations (12)–(15). These contributions might be relevant as values of joint angular velocity and acceleration as high as 2000 °/s and 20,000 °/s^2^, respectively, have often been recorded in high-velocity upper limb sport gestures [8,9,10,11,12]. Maple computing software (Maplesoft, Waterloo, ON, Canada) was used to perform numerical simulations.

## 3. Results

### 3.1. Condition 1

**Figure 2:**
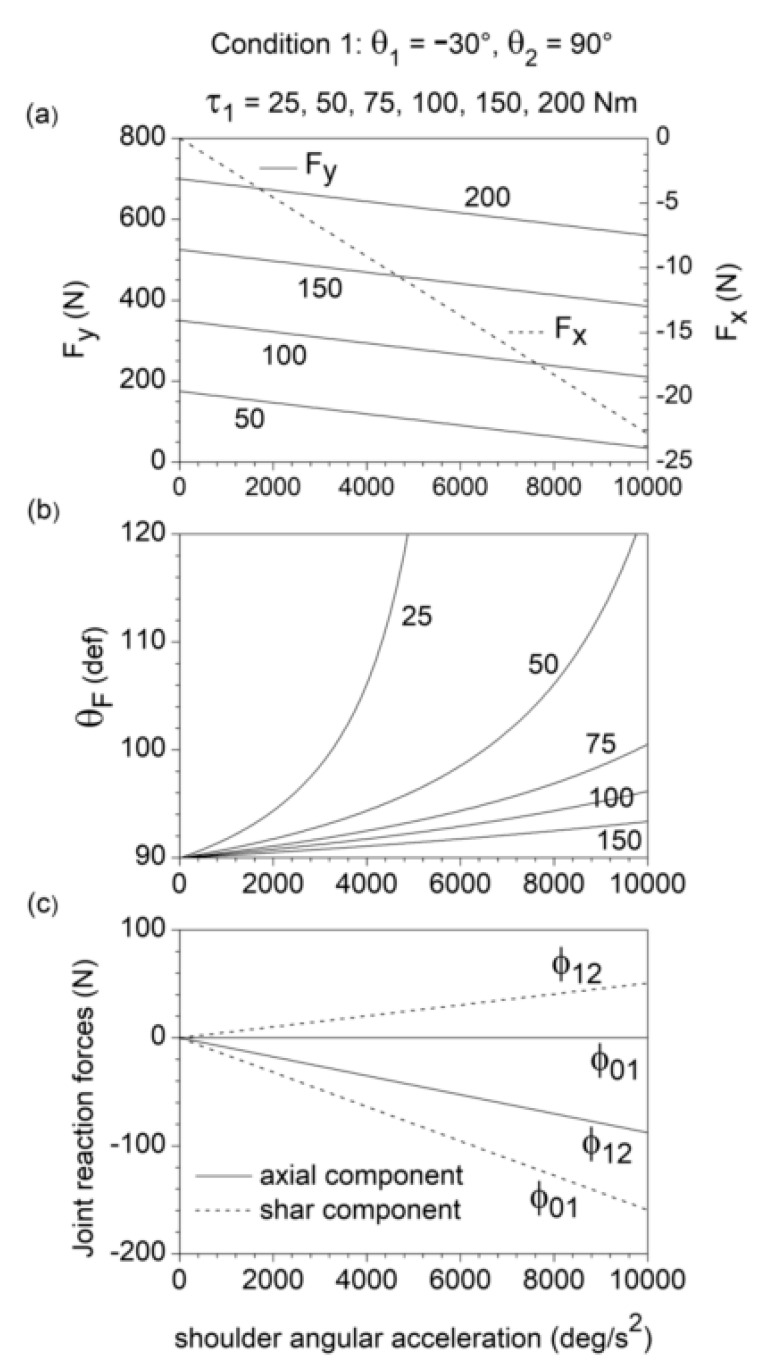
Dependence on the humerus angular acceleration ${\ddot{\theta }}_{1}$ of the components $F_{x}$ and $F_{y}$ of the endpoint force $\vec{F}$ (**a**), the direction $\theta_{F}$ of $\vec{F}$ (**b**), and the shear and axial components of the joint reaction forces ${\left(\phi_{0,1}\right)}_{\mathrm{axial}}$, ${\left(\phi_{0,1}\right)}_{\mathrm{shear}}$, ${\left(\phi_{1,2}\right)}_{\mathrm{axial}}$, and ${\left(\phi_{1,2}\right)}_{\mathrm{shear}}$ (**c**), for $\theta_{1}=-30{}^{\circ},\;\theta_{2}=90{}^{\circ}$ and ${\dot{\theta }}_{1}={\dot{\theta }}_{2}={\ddot{\theta }}_{2}=0$ (initial phase of upper limb push movement). In this condition, ${\ddot{\theta }}_{1}$ coincides with the shoulder angular acceleration. $F_{x}$, $F_{y}$, and $\theta_{F}$ are reported for different values of shoulder muscle torque $\tau_{1}$ and negligible elbow muscle torque ($\tau_{2}=0$ ). Only the selective effect of ${\ddot{\theta }}_{1}$ on the joint reaction forces was considered in (**c**).

An increase in $\tau_{1}$ yields a linear increase in the vertical component $F_{y}$ of the endpoint force $\vec{F}$ without affecting the horizontal component $F_{x}$ of $\vec{F}$ (Figure 2a). As expected, in the static condition (${\ddot{\theta }}_{1}={\dot{\theta }}_{1}=0$), $F_{x}=0$ and $\vec{F}$ is directed along the pointing axis ($\theta_{F}=90{}^{\circ}$). An increase in ${\ddot{\theta }}_{1}$ yields a linear decrease in both $F_{x}$ (which becomes negative) and $F_{y}$ (Figure 2a). As a result, the angle $\theta_{F}$ turns out to be a non-linear increasing function of ${\ddot{\theta }}_{1}$ and non-linear decreasing function of $\tau_{1}$, always greater than 90° for ${\ddot{\theta }}_{1}>0$ (Figure 2b). For the selected chain configuration ($\theta_{1}=-30{}^{\circ},\;\theta_{2}=90{}^{\circ}$), the values of $\theta_{F}$ may only range from 30° to 210°. In fact, for $210{}^{\circ}<\theta_{F}<360{}^{\circ}$ and $0{}^{\circ}<\theta_{F}<30{}^{\circ}$, the external resistance $\vec{R}$ acting on the endpoint ($\vec{R}=-\vec{F}$) would produce a horizontal shoulder flexion torque, changing the nature of the exercise from push to pull.

The shoulder acceleration ${\ddot{\theta }}_{1}$ produces negative shear force ${\left(\phi_{0,1}\right)}_{\mathrm{shear}}$ and axial force ${\left(\phi_{1,2}\right)}_{\mathrm{axial}}$, as well as positive shear force ${\left(\phi_{1,2}\right)}_{\mathrm{shear}}$. These three force components increase linearly in magnitude with ${\ddot{\theta }}_{1}$ (Figure 2c). Since ${\vec{\phi }}_{i-1,i}$ is the force exerted by the distal link (link *i*) on the adjacent proximal link (link *i* − 1) through the joint J*_i_*, this means that, due to the shoulder acceleration ${\ddot{\theta }}_{1}$, the humerus is subjected to a posterior shear force and the forearm to anterior shar force and axial compression. No axial force is induced by ${\ddot{\theta }}_{1}$ on the humerus.

### 3.2. Condition 2

**Figure 3:**
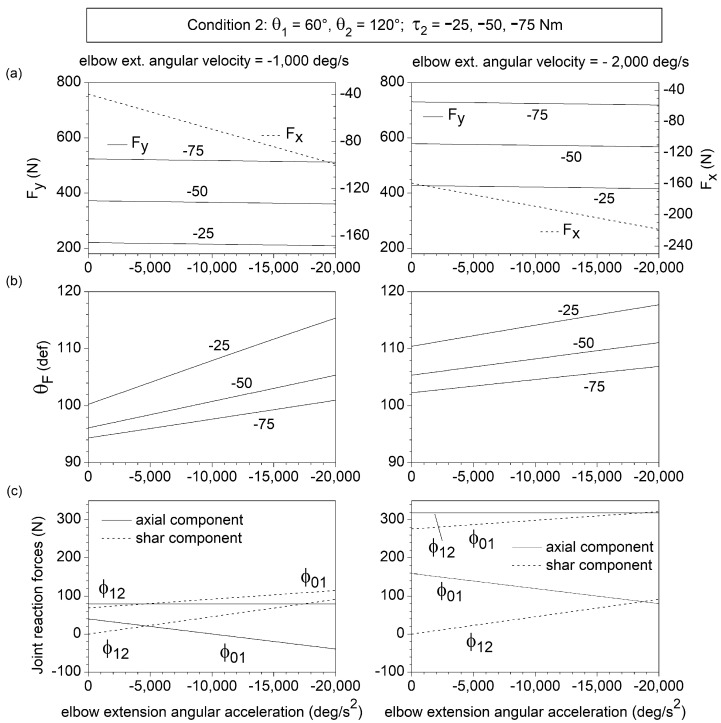
Dependence on the forearm angular acceleration ${\ddot{\theta }}_{2}$ of the components $F_{x}$ and $F_{y}$ of the endpoint force $\vec{F}$ (**a**), the direction $\theta_{F}$ of $\vec{F}$ (**b**), and the shear and axial components of the joint reaction forces ${\left(\phi_{0,1}\right)}_{\mathrm{axial}}$, ${\left(\phi_{0,1}\right)}_{\mathrm{shear}}$, ${\left(\phi_{1,2}\right)}_{\mathrm{axial}}$, and ${\left(\phi_{1,2}\right)}_{\mathrm{shear}}$ (**c**), for $\theta_{1}=60{}^{\circ},\;\theta_{2}=120{}^{\circ}$, ${\dot{\theta }}_{1}={\ddot{\theta }}_{1}\sim 0$, and ${\dot{\theta }}_{2}=-1000$ and −2000 deg/s (intermediate phase of upper-limb push movement). In this condition, ${\ddot{\theta }}_{2}$ coincides with the elbow angular acceleration. $F_{x}$, $F_{y}$, and $\theta_{F}$ are reported for different values of elbow muscle torque $\tau_{2}$ and negligible shoulder muscle torque ($\tau_{1}=0$ ). Only the selective effect of ${\ddot{\theta }}_{2}$ on the joint reaction forces was considered in Figure 2c.

**Figure 4:**
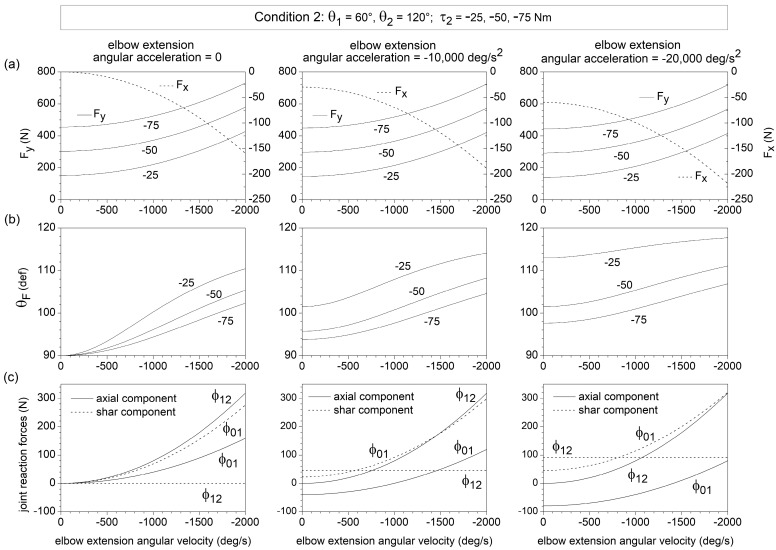
Dependence on the forearm angular velocity ${\dot{\theta }}_{2}$ of the components $F_{x}$ and $F_{y}$ of the endpoint force $\vec{F}$ (**a**), the direction $\theta_{F}$ of $\vec{F}$ (**b**), and the shear and axial components of the joint reaction forces ${\left(\phi_{0,1}\right)}_{\mathrm{axial}}$, ${\left(\phi_{0,1}\right)}_{\mathrm{shear}}$, ${\left(\phi_{1,2}\right)}_{\mathrm{axial}}$, and ${\left(\phi_{1,2}\right)}_{\mathrm{shear}}$ (**c**), for $\theta_{1}=60{}^{\circ},\;\theta_{2}=120{}^{\circ}$, ${\dot{\theta }}_{1}={\ddot{\theta }}_{1}\sim 0$, and ${\ddot{\theta }}_{2}=0$, −10,000, and −20,000 deg/s^2^ (intermediate phase of upper-limb push movement). In this condition, ${\dot{\theta }}_{2}$ coincides with the elbow angular velocity. $F_{x}$, $F_{y}$, and $\theta_{F}$ are reported for different values of elbow muscle torque $\tau_{2}$ and negligible shoulder muscle torque ($\tau_{1}\sim 0$ ). Only the selective effect of ${\dot{\theta }}_{2}$ on the joint reaction forces was considered in Figure 2c.

An increase in the elbow extensor torque $\tau_{2}$ from −25 Nm to −75 Nm enhances $F_{y}$ without affecting $F_{x}$ (Figure 3a and Figure 4a), thus, decreasing $\theta_{F}$ (Figure 3b and Figure 4b). $\theta_{F}$ ($F_{x}$) turns out to be an increasing (decreasing) function of both $\left|{\dot{\theta }}_{2}\right|$ and $\left|{\ddot{\theta }}_{2}\right|$ (negative values of ${\dot{\theta }}_{2}$ and ${\ddot{\theta }}_{2}$ correspond to elbow extension angular velocity and acceleration, respectively). The $F_{y}$ component is slightly affected by ${\ddot{\theta }}_{2}$ (Figure 3a) but displays a non-liner increasing dependence on $\left|{\dot{\theta }}_{2}\right|$ (Figure 4a).

The ${\left(\phi_{0,1}\right)}_{\mathrm{shear}}$ and ${\left(\phi_{1,2}\right)}_{\mathrm{axial}}$ components reach values as high as 300 N at the higher values of elbow extension angular velocity and acceleration (Figure 3c and Figure 4c). This corresponds to an anterior shear force acting on the humerus and an axial forearm traction. The forearm also experiences an anterior shear force (${\left(\phi_{1,2}\right)}_{\mathrm{shear}}>0$), which is independent of ${\dot{\theta }}_{2}$ and enhanced by $\left|{\ddot{\theta }}_{2}\right|$, and reaches a maximum magnitude of approximately 100 N. The elbow angular velocity and acceleration have opposite effects on the axial force ${\left(\phi_{0,1}\right)}_{\mathrm{axial}}$ acting on the humerus: ${\left(\phi_{0,1}\right)}_{\mathrm{axial}}$ is of traction type (positive) at high angular velocity and low angular acceleration of elbow extension and of compressive type (negative) at low angular velocity and high angular acceleration of elbow extension.

## 4. Discussion

This study provides a dynamic model for a planar two-link chain controlled by single-joint and two-joint muscles. Equations (9)–(11) fully characterize the chain endpoint force $\vec{F}$ in relation to the chain configuration, the link angular velocities and accelerations, and the joint torques developed by the single-joint and two-joint muscles. Likewise, Equations (12)–(15) determine the axial and shear components of the joint reaction force acting on the joints, given the chain configuration, the link angular velocities and accelerations, and the forces of the single-joint and two-joint muscles. These equations were used to analyze upper-limb ballistic push movements against resistance in the transverse plane, focusing on the dynamic effects of the shoulder and elbow angular acceleration in the initial phase of the movement and of the forearm angular velocity and acceleration in the subsequent phase of the movement.

The results highlight that the shoulder and elbow angular velocity and acceleration influence the direction of the endpoint force $\vec{F}$. In the static condition, the shoulder muscle torque produces endpoint force directed along the forearm axis, while the elbow muscle torque produces endpoint force along the radial axis connecting the shoulder to the endpoint [4]. In both chain conditions examined (see Section 2), this corresponds to a static endpoint force $\vec{F}$ directed along the *y*-axis $\left(\theta_{F}=90{}^{\circ}\right)$. The numerical simulation outcomes prove that a gradual increase in shoulder flexion angular acceleration in the initial phase of the movement, and in elbow extension angular velocity and acceleration in the subsequent phase of the movement, induce a progressive medial deviation in the $\vec{F}$ direction, with a shift in $\theta_{F}$ from 90° to 120° within the domain of interest of the values of the kinematic variables (Figure 2b, Figure 3b and Figure 4b). This information may have useful practical implications, as the optimization of the endpoint force direction constitutes a critical performance factor in pushing and throwing sport activities [25].

The link angular velocities and accelerations also significantly affect the value of specific axial and shear components of the joint reaction forces acting on the shoulder and elbow (Figure 2c, Figure 3c and Figure 4c). The shear force acting on the humerus (parallel to ${\hat{w}}_{1}$), produced by the humerus angular acceleration, is transmitted to the forearm through the elbow joint. This force acting on the forearm remains of the shear type (parallel to ${\hat{w}}_{2}$) if the elbow is extended, becomes axial type (parallel to ${\hat{u}}_{2}$) if the elbow is flexed at 90°, and gives rise to both axial and sheer forearm forces when the elbow is at 120° of flexion (as in the examined condition 1), with a prevalence of the axial component (Figure 2c). This simple example points out that the chain configuration plays a critical role in the transmission of axial and shear joint forces along the chain. Most importantly, the combined contribution of the elbow extension angular velocity and acceleration may give rise to shoulder shear forces and elbow traction forces as high as 300 N (Figure 4c). Advanced 3D models that consider the periarticular soft tissues and the complex geometry of the articular surfaces of the shoulder and elbow are necessary to estimate how the axial and shear joint forces are distributed among the different joint structures.

Equations (9)–(11) express the endpoint force components $F_{x}$ and $F_{y}$ as a function of the joint torques $\tau_{1}$ and $\tau_{2}$ and the chain kinematics ($\theta_{1},\;\theta_{2},\;{\dot{\theta }}_{1},{\dot{\theta }}_{2},{\ddot{\theta }}_{1},$ and ${\ddot{\theta }}_{2})$. However, these equations can also be used to determine $\tau_{1}$ and $\tau_{2}$ from the values of the variables $\theta_{1},\;\theta_{2},\;{\dot{\theta }}_{1},{\dot{\theta }}_{2},{\ddot{\theta }}_{1},{\ddot{\theta }}_{2},\;F_{x}$, and $F_{y}$ measured experimentally, for example, by a motion analysis system and load cells. This, in conjunction with electromyographic (EMG) measurements, would provide useful information on muscle function during two-joint exercises involving fast movements and external resistance.

The main limitation of this study concerns the kinematic conditions of the chain used as input parameters in the model, as defined by Equations (9)–(15). The values of these input parameters were not measured directly but selected according to the results of previous experimental studies reporting the kinematic patterns recorded during upper limb ballistic movements [8,9,10,11,12]. Overall, these previous studies examined groups of high-level athletes involved in different sports activities that were performed in specific sports environments. For this reason, we believe that the selected conditions refer to actual critical phases of upper limb ballistic movements that are of particular interest in sports and exercise science. Importantly, the explicit analytical formulation of the general Equations (9)–(15) enables the analysis of any other chain condition of specific interest.

Another limitation stems from the analysis of the shear and axial components of the joint reaction forces (Figure 2, Figure 3 and Figure 4). Only the contribution of the link angular velocities and accelerations was analyzed. The contribution of the resistance $\vec{R}$ ($\vec{R}=-\vec{F})$ can be immediately derived from Equations (12)–(15). Conversely, the determination of the contribution of the force of each single muscle in the chain would require knowledge of the muscle line of action in the specific chain configuration. The real magnitude of a muscle force can be estimated from the muscle architecture parameters and the degree of muscle activation with the use of EMG-driven musculoskeletal models and optimization procedures [26,27,28,29,30]. However, this is far beyond the scope of the present study. Nevertheless, for any set of values for the line of action and magnitude of the muscle forces, Equations (12)–(15) determine the contribution of the force of each muscle to the shear and axial components of the reaction force acting on each joint in the chain.

The intersegment dynamic model defined by Equations (9)–(15) was applied to upper limb ballistic movements in the transverse plane. Notably, however, the model can also be applied to any planar upper or lower limb movement by simply considering the components of the weights of the links in the plane of movement. Furthermore, the contribution of the joint angular velocity and acceleration to the axial and shear components of the joint reaction forces (Figure 2c, Figure 3c and Figure 4c) does not depend on the plane of movement. Therefore, the results of the present study can be used to quantify the axial and shear joint loads during several athletic and sport ballistic actions, including kicking a ball, punching, shot putting, explosive bench press throw, medicine ball throws, and overhead throwing.

## 5. Conclusions

The dynamic Equations (9)–(15) provide the closed-form analytical expressions of the endpoint force, and the shear and axial components of the joint reaction forces, for a planar two-link musculoskeletal chain controlled by single-joint and two-joint muscles. Numerical simulations highlight that the shoulder and elbow angular velocities and accelerations typically reached during ballistic sport movements can considerably affect the endpoint force direction and the shear and axial joint loads. This information can help estimate the mechanical stress acting on specific joint structures during explosive/ballistic sport movements and power-oriented exercises. It also helps in optimizing the endpoint force direction to improve the performance during these activities.

## Data Availability

The data that support the findings of this study are available from the corresponding author upon request.
